# Echocardiographic and laboratory findings in coronary slow flow phenomenon: cross-sectional study and review

**DOI:** 10.1186/s12872-021-02044-z

**Published:** 2021-05-05

**Authors:** Mir Hosein Seyyed Mohammadzad, Kamal Khademvatani, Salar Gardeshkhah, Amin Sedokani

**Affiliations:** Cardiology Department, Medical Faculty, Urmia University of Medical Sciences, 17 Shahrivar St., 571478334 Urmia, Iran

**Keywords:** Coronary angiography, Echocardiography, Coronary slow-flow, Laboratory findings

## Abstract

**Background:**

Coronary sow-flow phenomenon (CSFP) is defined as slow passage of the contrast injected into the coronary arteries without distal obstruction of the vessel.

**Methods:**

The present study was a cross-sectional, descriptive-analytical study performed at the Seyed-al-Shohada Heart Center during 2018–2019. The eligible patients based on the inclusion and exclusion criteria were divided into the study group showing the CSFP and the control group with normal epicardial coronary arteries.

**Results:**

The study included 124 patients. 67.9% of the study group and 39.4% of the control group were male patients (p-value = 0.001), and the mean patient age was 52.18 ± 12.55 and 51.77 ± 10.36 years in the study and control groups, respectively (p-value = 0.18). The study group had a significantly higher BMI than the control group (p < 0.05) and also a higher prevalence of smoking and hypertension. The variables of ALC, Hct, Plt, MPV, RDW, Cr, triglyceride, TC, and LDL, were higher in the study group. Given the echocardiographic findings, the mean E wave was significantly lower in the study group, while the control group had a significantly higher GLS (p-value = 0.01). Also, left anterior descending artery was the most common artery involved with CSFP.

**Conclusion:**

The CSFP was significantly more common in men, smokers, hypertensive patients, and patients with high BMI. Moreover, these patients had significantly higher platelet count, MPV, LDL, and FBS. Some other laboratory variables were also higher in these patients. Given the echocardiographic findings, mild diastolic dysfunction and low GLS were also observed in the study group.

## Background

The Coronary Slow-Flow Phenomenon (CSFP or cardiac syndrome Y) is defined as a delay in the distal contrast filling of a normal or almost normal coronary artery during angiography. This phenomenon can be seen in one or all of the coronary arteries [1–3]. The etiology is unknown; however, histological findings include myofibrillar hypertrophy, microvascular muscular thickening and swelling, endothelial degradation, and vascular lumen narrowing [4, 5]. The CSFP can be diagnosed using the Thrombolysis in Myocardial Infarction (TIMI) Flow Grade or the Corrected TIMI Frame Count (CTFC) [6, 7]. The phenomenon is observed in 1–7% of all the angiographies. 4% of the patients with unstable angina have CSFP as well. Moreover, it is more common in young male patients and smokers [5, 8]. The affected patients suffer from recurrent chest pain, frequent hospitalization, and repeated cardiac catheterization. Most importantly, some life-threatening arrhythmias (torsades de pointes) have been reported in these patients due to increased QT dispersion. Also, CSFP can lead to myocardial ischemia and subsequent Acute Coronary Syndrome (ACS) [9–11].

Similar to the Tissue Doppler Imaging (TDI), Two-dimensional (2D) Speckle Tracking Echocardiography (STE) is a new imaging modality used for offline calculation of myocardial velocities and ventricular deformation parameters such as the Strain Rate (SR). Also, Global Longitudinal Strain (GLS) is a robust, valid, and reproducible technique to assess the left ventricular deformation during echocardiography. It seems that GLS is a sensitive method for subclinical myocardial abnormality diagnosis and can analyze and treat regional and generalized wall defects [12, 13]. According to recent studies, determining the GLS using the STE method can reveal the slightest wall changes in patients with normal left ventricular Ejection Fraction (EF) [14].

The present study intended to investigate the echocardiographic and laboratory findings in the patients showing CSFP in angiography.

## Material and methods

The present study was a cross-sectional, descriptive-analytical study performed at Seyed-al-Shohada Heart Center, Urmia, West Azerbaijan Province, Iran. The study had a 1-year duration (August 2018 to August 2019) and included 124 patients. The eligible patients based on the inclusion and exclusion criteria were divided into two groups: the study group, including the patients with CSFP, and the control group, including the patients with normal epicardial coronary arteries (NECA). The participants were the patients who had undergone Coronary Angiography (CAG) due to one or more of these reasons: low-threshold typical angina pectoris along with atherosclerosis risk factors such as diabetes mellitus, hypertension, etc.; ECG changes or Myocardial Perfusion Imaging (MPI) findings positive for Coronary Heart Disease (CHD); or hospital admission to rule out ACS. Moreover, they had a normal ventral systolic function.

Patients underwent laboratory testing and echocardiography. The demographics, such as age and gender; clinical data on the underlying diseases; laboratory and paraclinical findings, such as WBC, Hb, Plt, MPV, RDW, Cr, FBS, TG, TC, LDL, and HDL; echocardiographic findings, such as the arteries involved with CSFP; and other data of the participants were recorded in a standard, pre-prepared checklist.

In the present study, a normal lipid profile was considered as LDL < 100 mg/dL, HDL ≥ 50 mg/dL for women or HDL ≥ 40 mg/dL for men, and total cholesterol < 200 mg/dL. Diabetes mellitus was defined as a previous history of the disease, having an FBS ≥ 126 mg/dL twice along with clinical symptoms, or having a HbA_1_C ≥ 6.4%.

Angiography was used for CSFP diagnosis and patient evaluation. The CTFC score (CTFC > 27 was considered as CSFP) and TIMI Grade Flow (TIMI-2-Flow was considered as CSFP) were used for quantitative and qualitative assessment of coronary blood flow, respectively [6, 8]. The patients were fully explained about the study goals and course, and the data confidentiality was ensured. Then, they gave informed consent. Laboratory and echocardiographic investigations were performed before the CAG. The angiography was performed using the femoral artery; however, the radial artery was used in hypertensive patients or those with a history of femoral artery problems. Also, the GLS was determined by an echocardiologist during the primary echocardiography.

Exclusion criteria included the patients with a previous history of CHD, such as a history of stent implantation, CABG surgery, or evidence of atherosclerotic coronary stenosis in a previous angiography; any significant valvular heart disease; pulmonary hypertension; Chronic Obstructive Pulmonary Disease (COPD); a Blood Pressure (BP) < 90/60 mmHg, a systolic BP > 180 mmHg, or a diastolic BP > 100 mmHg during the angiography; proven myocarditis; and atrioventricular conduction disorders. Also, no case of Myocardial Infarction with Non-Obstructive Coronary Arteries (MINOCA) was observed in the study.

### Data analysis

The data were described using the statistical indices of mean, frequency, and percentage. The qualitative variables, such as smoking status, gender, and family history, were described using the percentage, while the quantitative variables, including height, BMI, and laboratory findings, were described using the mean ± SD. Depending on the normality of data distribution, the Student's t-test (for independent samples) or the Mann–Whitney U test were used to investigate the quantitative variables, while the qualitative variables were analyzed using the chi-squared test. Data analysis was performed using the SPSS software version 26. The significance level was considered as P < 0.05 for all the comparisons.

## Results

### Gender

The present study included 124 patients. The study group included 67.9% (n = 36) male and 32.1% (n = 17) female patients, while the control group included 39.4% (n = 26) male and 60.6% (n = 43) female patients. According to the test results, there was a significant relationship between CSFP and gender, in a way that the study group had a significantly higher percentage of male patients than the control group (p = 0.001).

### Age

The mean patient age was 55.12 ± 18.52 with an age range of 26–74 in the study group, while it was 51.77 ± 10.36 with an age range of 27–72 in the control group. Therefore, there was no significant inter-group difference in the mean patient age (p = 0.18).

### Family history of CHD

According to our results, 35.8% of the patients in the study group and 21.1% of the patients in the control group had a positive family history of CHD; however, the intergroup difference was not significant (p = 0.10).

### BMI

The mean BMI of the study group was 28.13 ± 2.28 kg/m^2^ with a BMI range of 23–33, while it was 24.58 ± 1.64 kg/m^2^ with a BMI range of 21–29 in the control group was. Therefore, the study group patients had a significantly higher BMI than those of the control group (p < 0.001).

### Smoking

62.2% (n = 33) of the patients in the study group were active smokers, 18.3% (n = 13) of the control group patients were active smokers. Therefore, the number of active smokers was significantly higher in the study group than in the control group (p < 0.001).

### Hypertension

58.5% and 25.4% of the patients in the study and control groups had hypertension, respectively. Therefore, hypertension was significantly more prevalent in the study group than in the control group (p < 0.001).

### Diabetes, dyslipidemia, and chronic kidney disease

There were no significant inter-group differences in the variables of diabetes mellitus, dyslipidemia, and Chronic Kidney Disease (CKD) (p = 0.91, p = 0.49, and p = 0.65, respectively).

### Laboratory findings

The inter-group comparison of the laboratory parameters was performed using the independent t-test. According to the results, Absolute Neutrophil Count (ANC), Absolute Lymphocyte Count (ALC), Hct, Plt, MPV, RDW, Cr, TG, total cholesterol, and LDL were significantly higher in the study group than in the control group (Table 1).

**Table 1:** Laboratory and Echocardiographic findings among CSF and NECA groups (values ± SD)

Variable	CSF (study group)	NECA (control group)		P-value
Laboratory				
WBC	× 10^3^/ml^3^	7.13 ± 0.87	7.16 ± 1	0.23
Neutrophil	× 10^3^/ml^3^	4.03 ± 0.80	4.80 ± 0.92	**0.001**
Lymphocyte	× 10^3^/ml^3^	1.95 ± 0.52	1.30 ± 0.12	**< 0.001**
N/L Ratio^*^	–	2.36 ± 1.74	3.70 ± 0.73	0.90
Hemoglobin	gr/dL	15.39 ± 1.29	13.10 ± 1.14	0.121
Hematocrit	%	45.25 ± 3.62	38.77 ± 2.19	**< 0.001**
Platelet	× 10^4^/ml^3^	23.34 ± 3.82	17.46 ± 1.36	**< 0.001**
MPV	fL	13.10 ± 1.72	9.90 ± 1.10	**0.011**
RDW	%	13.21 ± 1.76	9.55 ± 1.06	**0.037**
Creatinine	mg/dL	1.17 ± 0.23	1.07 ± 0.14	**0.016**
BUN	mg/dL	16.09 ± 2.49	14.75 ± 1.77	0.144
FBS	mg/dL	108.60 ± 11.21	90.08 ± 9.45	0.143
Triglyceride	mg/dL	202.80 ± 48.51	131.84 ± 34.22	**0.001**
Total cholesterol	mg/dL	221.50 ± 49.89	144.37 ± 33.21	**0.001**
LDL	mg/dL	151.48 ± 34.28	103.34 ± 21.70	**< 0.001**
HDL	mg/dL	46.56 ± 6.56	52.91 ± 6.21	0.756
Echocardiography				
LVEF	%	54.52 ± 1.47	54.78 ± 1.01	**0.020**
IVSD	cm	1.02 ± 0.10	1.03 ± 0.08	**0.049**
LVEDD	cm	4.96 ± 0.14	5.00 ± 0.21	0.201
LVESD	cm	2.66 ± 0.15	2.68 ± 0.22	0.861
E wave	ms	0.63 ± 0.17	0.75 ± 0.14	**0.029**
A wave	ms	0.75 ± 0.12	0.73 ± 0.11	0.546
E/A ratio	–	0.85 ± 0.24	1.03 ± 0.20	0.733
DT	ms	179.94 ± 7.61	180.44 ± 5.54	0.093
LV systolic GLS	–	− 15.86 ± 0.91	− 18.59 ± 0.59	**0.010**

The bold values in P-value column, are significant as p-value < 0.05

*Neutrophil/Lymphocyte Ratio

### Echocardiographic findings

The inter-group comparison of the echocardiographic findings was performed using the independent t-test. According to the results, LVEF, IVSD, and E wave were significantly lower in the study group than in the control group (Table 1).

### Arteries involved

Of the arteries involved with CSFP in the present study, 46.8% were left anterior descending arterys (LADs), 27.9% were the Left Circumflex arteries (LCX), and 25.3% were the Right Coronary Arteries (RCA). Therefore, LAD was the most common artery involved with CSFP.

### Global longitudinal strain for LV function

**Fig. 1:**
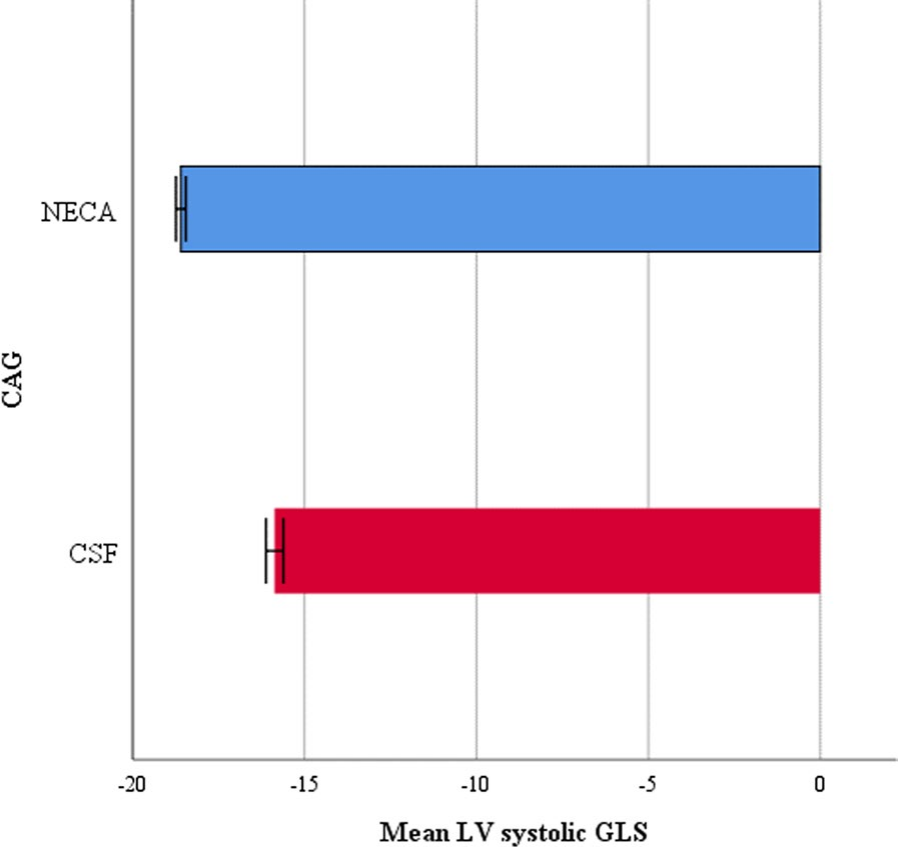
Comparison of the mean GLS of the left ventricle between the study group (n = 53) and the control group (n = 71) using the Student's t-test. Error Bar Confidence Interval: 95%

According to the independent t-test results, the mean LV systolic GLS was significantly higher in the control group than in the study group (p = 0.01, Table 1, Fig. 1).

## Discussion

The CSFP is diagnosed by the slow flowing of the contrast material during angiography in normal or almost-normal coronary arteries [1–3, 15]. This phenomenon can mimic the clinical manifestations of various conditions, including unstable angina, acute myocardial infarction, and ventricular tachycardia [16, 17]. The exact etiology and pathophysiology of the phenomenon are not yet understood; however, various hypotheses have been suggested by different studies, including endothelial dysfunction, microvascular dysfunction, early-stage atherosclerosis, myocardial fibrosis, metabolic disorders, and inflammatory disorders [17–23]. Histological investigations on the biopsies from CSFP patients have shown fibromuscular hyperplasia, medial hypertrophy, myointimal proliferation, endothelial edema, and small vessel degeneration [5, 18].

Recently, it has been reported that decreased adiponectin concentration and decreased paraoxonase activity, which are two important markers of endothelial dysfunction, are associated with CSFP [24]. Moreover, it has been shown the patients with CSFP have a decreased flow-mediated dilatation (FMD) of the brachial artery, indicating endothelial dysfunction [18]. Also, increased plasma levels of homocysteine and dimethylarginine have been reported in these patients, which is associated with decreased nitric oxide (NO) levels that may subsequently impair the endothelial function [19, 20].

Despite many studies, there is no certain explanation for the exact mechanism of CSFP [1–4, 13, 16].

According to the studies, metabolic syndrome, along with insulin resistance or impaired glucose tolerance, high cholesterol, high fasting glucose, and high BMI are more prevalent in patients with CSFP [21, 22].

Microvascular dysfunction is another potential cause of the phenomenon. It is observed in small, resistive vessels with a diameter lower than 400 μm. These vessels control the myocardial blood flow without apparent stenosis in the coronary epicardial arteries. This microvascular dysfunction observed in CSFP patients may be explained by fibromuscular hyperplasia, medial hypertrophy, myointimal proliferation, or endothelial edema [5]. Using the IVUS investigations, it was shown that the CSFP patients had an increased intimal thickness and diffuse coronary calcification. These findings suggest that CSFP can be a non-obstructive atherosclerotic disease; however, this hypothesis needs further investigation [23]. Moreover, CSFP patients are in an inflammatory state that manifests itself with increased inflammatory markers, such as CRP, IL-6, and WBC, and anatomical abnormalities of the coronary arteries (geometrical motions, coronary angles, bifurcation, etc.).

According to some studies, CSFP patients have a decreased LV systolic and diastolic function, while the RV function is not changed. These changes are detectable using the TDI/GLS method [17, 25]. Moreover, some other studies emphasizing the TDI/GLS evaluation of CSFP patients have reported an impaired LV systolic and diastolic function using the 2D-TDI (26, 27).

There have been various studies on the TDI/GLS evaluation; echocardiographic, clinical, and angiographic findings; laboratory predictors; and risk factors of the CSFP patients [26–29]. However, no comprehensive study has yet been conducted on the laboratory and echocardiographic findings of the patients with CSFP.

In our study, the percentage of male patients was significantly higher in the study group than in the control group, which was compatible with many similar studies reporting a higher prevalence of CSFP in men. However, our study and other similar studies found no relationship between CSFP and age [25, 28, 30–34]. Therefore, according to available evidence, age does not affect the pathophysiology of CSFP. However, more detailed data may change this conclusion. These data can be obtained by conducting further studies with longer study duration and frequent angiographic investigations. In terms of gender-related findings, gender-oriented pathophysiology has not yet been proposed. Therefore, it seems that these gender-related differences may be due to factors such as hormonal changes, stress levels, or different work conditions.

In the present study, a higher BMI was reported in patients with CSFP, and a BMI decrease could reduce the prevalence of CSFP. These findings were compatible with most other studies in this field, which found an independent relationship between CSFP and high BMI [28, 29, 31–33]. For example, Hawkins et al. on the North American population showed BMI as an independent predictor of CSFP [26]. However, a study by Sanati et al. reported the association of low BMI with CSFP [28]. This finding can be explained by the fact that obesity can lead to endothelial dysfunction. Therefore, it can play a role in the CSFP pathogenesis. Pontiroli et al. showed an improved endothelial system function following the gastric banding procedure and subsequent BMI decrease [27]. Although high BMI is a predictor of CSF, other studies are needed to find the improving effect of lowering BMI on the CSFP.

According to our results, smoking and hypertension were significantly more prevalent in the study group than in the control group, while no intergroup difference was found in the variables of diabetes mellitus, dyslipidemia, and family history of CHD. These findings were compatible with some studies that found no relationship between the CSFP and the variables of diabetes mellitus, hypertension, dyslipidemia, and family history of CHD [32, 35]. Moreover, some other studies found that CSFP and the variables of smoking and hypertension were significantly correlated [28, 30, 31, 34].

The CSFP pathophysiology or risk factors are not yet fully illustrated. Different studies on various populations have found different risk factors for this phenomenon. For example, a study performed in Australia reported smoking and male gender as the most important risk factors of CSFP [2], while according to another study in China, hyperuricemia, hyperglycemia, and high levels of High Sensitivity C-Reactive Protein (hsCRP), which had roles in endothelial dysfunction, were found to be the independent risk factors of CSFP [36]. Moreover, a study by Moazenzadeh et al. reported diabetes mellitus, hypertension, and opioid abuse as the main risk factors of CSFP [37]. It seems that the CSFP risk factors are different in various populations with different genetic backgrounds. It is very likely that one or more of these risk factors play roles in CSFP development by causing endothelial dysfunction.

The present study found significantly higher levels of ALC, Hct, Plt, MPV, RDW, Cr, TG, total cholesterol, and LDL in the CSFP patients than in the control group while ANC was significantly lower. Also, the two groups were not different in WBC and BUN. These findings were not compatible with some studies reporting lower levels of Cr, uric acid, Hct, MCV, and HDL in the CSFP patients, as well as no significant changes in WBC, Plt, FBS, and MPV [16, 28, 30]. According to a study by Ghaffari et al., total cholesterol, TG, Hb, Hct, ALC, Plt, platelet distribution, RDW, MPV, and FBS were significantly higher in the CSFP patients, while WBC, Cr, Absolute Monocyte Count (AMC), and ANC were not significantly changed.

It seems that patients with CSFP may have an underlying inflammatory state and endothelial dysfunction [31, 38]. A study by Narimani et al. on CSFP patients found high LDL and low HDL levels in these patients [32]. These findings suggest that the incidence of CSFP may be reduced by correcting the lipid profile through diet modification, physical activity, or medication. However, most aspects of the CSFP mechanism are not yet understood. For example, there are some other studies showing no significant changes in Hct, Plt, uric acid, HbA1C, homocysteine [33], general lipid profile, and other blood markers in CSFP patients [25]. However, there is a need for more research in this area.

Altun et al. found significantly higher levels of Cr and Hb in the CSFP patients, while no significant inter-group difference was found in MPV, RDW, and Neutrophil-to-Lymphocyte Ration (NLR). It has been shown that inflammation plays an important role in CSFP pathogenesis. However, the association of CSFP with inflammatory markers is still controversial [34, 39]. For example, some studies have found a strong association between RDW and inflammatory markers [40], while some other studies have found a relationship between high RDW and CSFP [41].

It seems that platelet dysfunction is also effective in CSFP pathogenesis because some studies have shown that these patients are more likely to have platelet aggregations than the control patients. MPV is a valuable marker to assess platelet dysfunction. It has been shown that this parameter has a significant positive correlation with CSFP [42–44].

Given the echocardiographic findings, we found a significantly lower E wave in the study group than in the control group. These findings were compatible with a study by Wang et al. that found significantly lower E wave and E/A ratio in the CSFP patients while no changes in A waves [30]. Moreover, there have been some other studies reporting left ventricular systolic and diastolic dysfunction in these patients [45, 46]. It is suggested that ischemic CHD can be the reason for this systolic and diastolic dysfunction [47–49]. However, we only observed a mild diastolic dysfunction in these patients, and there was significant difference in terms of EF, although they had higher GLS than the control group, which may indicate the physiological dysfunction of the left ventricle. Moreover, we did not find significant inter-group differences in LVESD, LVEDD, A wave, and E/A ratio, while a study by Narimani et al. found significant inter-group differences in LVESD, LVEDD, EF, E waves, A waves, E/A ratio, DT, and IVRT. They also reported that E and S waves of the lateral wall were lower in these patients [32]. There was also another study that found no difference in LVEF [30].

GLS measurement is a completely non-invasive method to diagnose and evaluate systolic and diastolic function. This parameter measures the myocardial movements in all directions, providing us with the total left ventricular tension at all angles (longitudinal, radial, and marginal) [50]. GLS is very sensitive in diagnosing left ventricular dysfunction at the onset of many myocardial pathological conditions [23, 51]. As previously stated, the mean GLS of the study group was significantly lower than the control group in the present study. This finding was compatible with a study by Wang et al. that reported a significantly lower level of left ventricular diastolic and systolic longitudinal traction in patients with the CSFP than in the control group [30].

However, Narimani et al. found no significant relationship between the CSFP and the systolic and diastolic longitudinal traction, showing that CSFP could not impair the systolic and diastolic longitudinal function [32]. Another study by Nurkalem et al. also showed that the left ventricular longitudinal strain was different between the CSFP and control groups [52]. Therefore, the presence of left ventricular dysfunction in CSFP is still controversial, so further studies are needed to elucidate this relationship. According to the literature, longitudinal traction impairment of the left ventricle occurs earlier than the marginal and radial traction impairments [53].

As the last finding, we found that LAD was the most common artery affected by CSFP, followed by LCX and RCA. This finding was compatible with a study by Yildiz et al. that found higher cases of CSFP in LAD [28]. There have also been similar studies reporting the highest occurrence of CSFP in the arteries LAD, LCX, and RCA [25, 30, 33]. The prevalence of CSFP can differ widely in different arteries depending on the technical and genetic contexts and also the variables involved in each study. However, LAD was still the most common artery involved in most studies.

## Conclusion

According to our findings, we concluded that the CSFP was significantly more common in male patients, smokers, patients with high BMI, and hypertensive patients. In terms of laboratory findings, ALC, Hct, Plt, MPV, RDW, Cr, triglyceride, TC, and LDL, were significantly higher in these patients. In terms of echocardiographic findings, these patients had mild diastolic dysfunction and decreased left ventricular GLS.

## Data Availability

The datasets used and/or analyzed during the present study are available from the corresponding author on reasonable request.
